# Natural Co-Exposure to *Borrelia burgdorferi* s.l. and *Anaplasma phagocytophilum*: Unraveling the Hematological Profile in Sheep

**DOI:** 10.3390/life13020469

**Published:** 2023-02-08

**Authors:** Labrini V. Athanasiou, Constantina N. Tsokana, Dimitris A. Gougoulis, Athanasia H. Tzivara, Anna Dedousi, Panagiotis D. Katsoulos

**Affiliations:** 1 Clinic of Medicine, Faculty of Veterinary Medicine, University of Thessaly, 43100 Karditsa, Greece; 2 Veterinary Research Institute, HAO-Demeter, 57001 Thessaloniki, Greece; 3 Clinic of Farm Animals, Faculty of Veterinary Medicine, Aristotle University of Thessaloniki, 54627 Thessaloniki, Greece

**Keywords:** *Anaplasma phagocytophilum*, *Borrelia burgdorferi* sensu lato, hematology, immunofluorescence, serology, sheep

## Abstract

The occurrence of co-infected hosts and questing ticks with more than one tick-borne pathogen—as in the case of *Anaplasma phagocytophilum* and *Borrelia burgdorferi* sensu lato—is expected in endemic regions. Their synergy—in terms of pathogenesis and disease severity—has been suggested previously in humans. Limited data exist on the clinicopathological alterations in co-infected sheep. In this study, we investigated the impact of *A. phagocytophilum* and *B. burgdorferi* s.l. seropositivity, alone and in combination, on the hematological parameters of naturally infected sheep. A complete blood count was performed, and indirect immunofluorescence assays were used to detect IgG antibodies against *A. phagocytophilum* and IgG and IgM antibodies against *B. burgdorferi* s.l. Single natural exposure to *B. burgdorferi* s.l. was characterized by low Packed Cell Volume (PCV) values and platelet (PLT) counts, while single exposure to *A. phagocytophilum* was characterized by low PCV values, low white blood cell (WBC) counts, and an increased risk for leukopenia and neutropenia. Co-exposure resulted in the most severe blood abnormalities; all the blood parameters decreased, and the sheep presented an increased risk for anemia. Our study showed that natural co-exposure to *A. phagocytophilum* and *B. burgdorferi* s.l. in sheep leads to more severe blood abnormalities and enhances the pathogenic processes. More studies are needed to clarify the possible background mechanisms.

## 1. Introduction

Several tick species infest sheep worldwide, with *Dermacentor marginatus*, *Haemaphysalis punctata*, *Ixodes ricinus,* and *Rhipicephalus bursa* being the dominant ones in Europe [1]. Ticks act both as direct blood-feeding parasites and as vectors of a range of pathogens. Tick infestation in sheep can have a severe impact on animal welfare, health, and production indices [2]. The different tick-borne pathogens (TBPs) that are transmitted to sheep, such as *Anaplasma ovis*, *A. phagocytophilum*, *Borrelia burgdorferi* sensu lato, *Babesia* spp., and Louping-ill virus, follow the distribution of their vectors and sheep populations [3].

Concurrent infection with more than one TBP is common. It can occur when ticks—each one infected with a different pathogen, or ticks that are co-infected with two or more TBPs at the same time—feed on a host. Several infected ticks can feed on the same host simultaneously or at different times [4]. In the case of *A. phagocytophilum*, which shares a common vector with *B. burgdorferi* s.l., the occurrence of co-infected hosts and questing ticks is expected in endemic regions [5,6].

*Anaplasma phagocytophilum* is an obligate intracellular bacterium that replicates mainly in the neutrophils of animals and humans and is transmitted through hard ticks of the genus *Ixodes* [3,7]. Infection has been reported in a wide variety of wild and domestic animals worldwide [8], but persistent infection has been reported in sheep and rodents, which are considered to serve as reservoir hosts, facilitating further spread of the pathogen [9].

In domestic ruminants, the disease caused by *A. phagocytophilum* is known as tick-borne fever (TBF). Although they may escape diagnosis as an invisible infection, sheep present fever, anorexia, conjunctivitis, abortion, reduced milk yield and weight gain in young animals, impaired spermatogenesis in males, and immunosuppression [10,11]. TBF is seldom fatal unless complicated by other infections; the affected animals are vulnerable to secondary infections, such as tick pyaemia caused by *Staphylococcus* spp.—a wasting and crippling condition [3]. In humans, *A. phagocytophilum* is the causative agent of human granulocytic anaplasmosis (HGA), which is characterized by headaches, general joint and muscle pain (myalgia), and fatigue [12]. Although anaplasmosis is considered the most widespread tick-borne infection in animals in Europe and shows an increasing trend [13], the human cases are not frequent. They are most likely underestimated due to the nonspecific clinical signs of the disease (flu-like symptoms) [12].

The diagnosis of anaplasmosis in animals is based on the detection of inclusions within the infected leukocytes using microscopic examination (mainly during the acute phase of the disease), the DNA of *A. phagocytophilum* using molecular methods, and IgG antibodies using serological methods. The indirect immunofluorescence antibody test (IFA), the enzyme-linked immunosorbent assay (ELISA), and the complement fixation test (CF) are the most frequently used serological methods [7,14]. Based on molecular analysis, several lineages and ecotypes have been reported worldwide for *A. phagocytophilum*; they exhibit a high degree of genetic diversity, host tropism, and variation in pathogenicity [15].

The seroprevalence of *A. phagocytophilum* in sheep in Europe is high: 36.0% in Norway [16], 47% in the Czech Republic (with even 100% seropositivity in a flock in that study) [17], to 55% in Norway [18], and 60.5% in Sweden [19]. However, serologic cross-reactivity with other *Anaplasma* spp., e.g., *A. ovis*, needs to be considered [20]. Data on the seroprevalence of *A. phagocytophilum* in sheep in Greece are lacking; a recent study showed that sheep in Greece are exposed and/or infected with this pathogen and investigated the alterations caused by the presence of antibodies and/or the antigen of *A. phagocytophilum* in the blood cell count and morphology in sheep [21], while the first clinical case of ovine anaplasmosis in Greece caused by *A. phagocytophilum* was reported in 2011 in a 10 month old tick-infested ram in Thessaloniki, Northern Greece, by Giadinis et al. [22].

Seroprevalence studies in humans in Greece are limited, but they revealed the occurrence of antibodies against *A. phagocytophilum* in healthy individuals: 7.3% in rural areas of northern Greece [23] and 21.4% in healthy blood donors in Crete [24], where the first cases of severe HGA were reported as well [25]. Up until now, the only fatal case of HGA in Greece concerned a man living in Athens who had been exposed to an *A. phagocytophilum*-infected tick while gardening [26]. Molecular evidence of *A. phagocytophilum*-infected *I. ricinus* ticks collected from goats in northern Greece had been provided earlier in the study of Kachrimanidou et al. [27]. Recently, serological studies in dogs in Greece showed a low prevalence of antibodies against *Anaplasma* spp.: 6.2% in apparently healthy dogs in the 66 municipalities of the country [28] and 2.3% in dogs living on the Greek islands in the Ionian and Aegean seas [29].

The *B. burgdorferi* s.l. complex includes the etiological agent of Lyme disease and currently comprises more than 20 named and proposed genospecies that are associated with different clinical manifestations of the disease [30]. The pathogen is transmitted by ticks of the genus *Ixodes*, and it infects a great variety of animal species worldwide; cattle, sheep, horses, dogs, and rats serve as reservoirs with varying clinical symptoms [31,32]. Migratory sea birds are of major importance as well because they can transfer ticks over very long distances and thus contribute to the spread of borreliae worldwide [33].

Clinical relevance of *B. burgdorferi* s.l. in sheep is questionable. Experimental infections failed to produce any symptoms [34], but clinical manifestations have been suspected in this species; a few cases of Lyme borreliosis (LB) have been reported in sheep presenting lameness, anorexia, and poor body condition [35]. Sheep seroconvert when exposed to the pathogen and may therefore be considered as sentinels [10].

The reported seroprevalences range from 0% in healthy sheep in central Sweden to 84.6% in lambs with arthritis on the island of Gotland, Sweden [36]. In asymptomatic sheep, the highest seroprevalence (56.5%) was recorded in France [37]. Recently, a study in sheep reported a seropositivity of 23.6% in Greece; anemia, thrombocytopenia, and a normal or decreased leukocyte count were the main hematological features observed in seropositive sheep [38].

In Greece, there is only one country-wide study reporting a very low seroprevalence of 3.3% in randomly selected healthy Greek Navy recruits [39], while no records of human disease have been described so far [40]. Seroepidemiological studies have been conducted mainly in dogs [28,29,41]. Although differences in these studies may reflect different serological methods employed and the geographical area of the sampled dogs’ origin, seroprevalence seems to be quite low in the species: 0.1% in apparently healthy dogs in a country-wide study [28] and 2.2% in mainland Greece [41]. Notably, co-exposure to *Leishmania infantum*, *Dirofilaria immitis* [41], *Ehrlichia* spp., and *Anaplasma* spp. [28] has been identified in the sampled dogs.

Limited data exist on the clinicopathological alterations occurring in *Anaplasma* and/or *Borrelia*-infected sheep following both experimental and natural infection. To the authors’ best knowledge, there is no data available on the hematological profile of sheep that have been naturally exposed to both agents. Interestingly, a previous study in humans co-infected with *A. phagocytophilum* and *B. burgdorferi* sensu stricto showed a synergy between these two pathogens in terms of pathogenesis and disease severity [42].

The objective of this study was to assess the impact of *A. phagocytophilum* and *B. burgdorferi* seropositivity, alone and in combination, on the hematological variables of naturally infected sheep. A complementary goal was to evaluate the seropositivity against *A. phagocytophilum* and/or *B. burgdorferi* as potential risk factors for the presence of anemia, leukopenia, neutropenia, lymphopenia, and thrombocytopenia.

## 2. Materials and Methods

### 2.1. Animal Selection Criteria

A total of 450 sheep of the Chios breed from 6 farms in Greece were enrolled in this study. The inclusion criteria were (a) tick infestation and (b) deworming treatment performed in the last 2 months. Animals were excluded based on (a) the presence of other ectoparasites such as fleas and lice and (b) serological (employing an indirect immunofluorescent assay for the detection of antibodies) or cytological evidence (on whole blood as well as on Giemsa-stained buffy coat smears) of other concurrent tick-borne infections (*Babesia* sp., *Theileria* sp., *Rickettsia* sp.).

### 2.2. Blood Sampling

Blood samples were collected by jugular venipuncture into plein and EDTA-coated vacuum tubes (BD, Franklin Lakes, NJ, USA) for serum retrieval and whole blood, respectively. All samples were transferred on ice, avoiding direct contact with the tubes, to the Diagnostic Laboratory, Faculty of Veterinary Medicine, School of Health Sciences, University of Thessaly, Greece, within 24 h.

### 2.3. Complete Blood Count

The packed cell volume (PCV) value was assessed by the microhematocrit method [43]. The red cell column height formed after centrifugation of the tube is representative of the PCV value.

Blood smear microscopy was performed on each sample. Blood smear preparations were fixed using methanol and stained with Giemsa. Quantitative assessment of white blood cells (WBCs) and platelets (PLTs) was performed as previously described [14]. Briefly, the number of WBCs and PLTs was microscopically assessed, and a previously validated equation was used to convert the detected value to the corresponding value of the ADVIA 120 hematology analyzer [14]. A differential WBC count was performed manually on 200 leukocytes. The interpretation of the values was based on previously published reference intervals [44].

### 2.4. Indirect Immunofluorescence Antibody (IFA) Assay

Serum separation was performed after blood clotting by centrifugation of the tube at 300× *g* for 10 min. Serum supernatant was transferred into plastic vials (Eppendorf Tubes^®^, Eppendorf AG, Hamburg, Germany) and stored at −20 °C pending analysis. An IFA assay was performed for the detection of IgG and IgM antibodies against *B. burgdorferi* s.l. and IgG antibodies against *A. phagocytophilum* in serum as described previously [21,38]. Titers ≥ 1:64 for *B. burgdorferi* s.l. and ≥1:40 for *A. phagocytophilum* were defined as positive.

### 2.5. Groups

Based on the results of the antibody detection assays, the sampled animals were allocated into seven groups (Table 1); (a) BorIgM+: sheep with IgM against *B. burgdorferi*; (b) BorIgG+: sheep with IgG against *B. burgdorferi;* (c) BorIgG&M+: sheep with IgM and IgG against *B. burgdorferi;* (d) An+BorIgG+: sheep with IgG against *A. phagocytophilum* and IgG against *B. burgdorferi;* (e) An+BorIgG&M+: sheep with IgG against *A. phagocytophilum* and IgM and IgG against *B. burgdorferi*; (f) An+: sheep with IgG against *A. phagocytophilum;* and (g) seronegative animals (control group).

### 2.6. Data Analysis

The obtained data were analyzed using the statistical program JASP 16.4. Linear regression models were built to evaluate the impact of seropositivity (positive-negative) for *A. phagocytophilum*, *B. burgdorferi,* and their combinations on PCV, WBCs (total and differential WBC counts), and PLTs [45].

The model used for each parameter evaluated to quantify this impact is described below:

Y_i_ = μ + BM + BG + BGM + ABG + ABGM + An

where:

Y_i_ = values of the hematological parameter evaluated, μ = intercept, BM = fixed effect of IgM seropositivity for *B. burgdorferi* (2 levels; 0 = negative and 1 = positive), BG = fixed effect of IgG seropositivity for *B. burgdorferi* (2 levels; 0 = negative and 1 = positive), BGM = fixed effect of IgG and IgM seropositivity for *B. burgdorferi* (2 levels; 0 = negative and 1 = positive), ABG = fixed effect of IgG seropositivity for *B. burgdorferi* and IgG seropositivity for *A. phagocytophilum* (2 levels; 0 = negative and 1 = positive), ABGM = fixed effect of IgG and IgM seropositivity for *B. burgdorferi* and IgG seropositivity for *A. phagocytophilum* and An = fixed effect of IgG seropositivity for *A. phagocytophilum.*

Logistic regression models were also built to investigate these seropositivities as potential risk factors for the presence of anemia, leukopenia, neutropenia, lymphopenia, and thrombocytopenia. The enter method was used in all models, and the probability level was set at *p* ≤ 0.05.

## 3. Results

A total of 450 animals were tested. Out of the 248 samples meeting the inclusion criteria, 27 were assigned to BorIgM+ group, 48 to BorIgG+ group, 18 to BorIgG&M+ group, 27 to An+BorIgG+ group, 25 to An+BorIgG&M+ group, 16 to An+ group, and 87 to the group of seronegative animals for both pathogens (controls). Anemia was detected in 94 animals, thrombocytopenia in 99, leukopenia in 50, neutropenia in 45, and lymphocytopenia in 27.

Linear regression models revealed that seropositivity for *Borrelia* and/or *Anaplasma* was associated with a significant reduction (*p* < 0.05) of PCV (Table 2). The highest reduction was recorded in seropositive animals for An+BorIgG&M+, followed by those for BorIgG&M+. The lowest reduction was detected in An+ animals. BorIgM+ and An+ seropositivity had no significant effect on PLT counts (*p* > 0.05); however, BorIgG+, BorIgG&M+, An+BorIgG+, and An+BorIgG&M+ seropositivity were associated with significantly lower PLT counts (*p* < 0.001; Table 2). The highest significant reduction was detected in An+BorIgG&M+ and BorIgG&M+ animals, and the lowest in BorIgG+.

As shown in Table 3, except for BorIgG+, *Borrelia* and/or *Anaplasma* seropositivity were associated with a significant reduction of WBC counts as well (*p* < 0.05). An+BorIgG&M+ had the highest significant impact on WBC counts, and BorIgM+ the lowest. The same trend was also observed for neutrophil counts (*p* < 0.05), but BorIgG+ seropositivity was associated with a significant increase (*p* < 0.05) of these cells. BorIgG&M+, An+BorIgG+, and An+BorIgG&M+ seropositivity were associated with a significant reduction in lymphocyte counts (*p* < 0.05), whereas the other seropositivities had no significant impact (*p* > 0.05). Monocyte counts were significantly reduced (*p* < 0.05) only in BorIgM+, An+BorIgG+, and An+BorIgG&M+ seropositive animals (Table 4). Eosinophil counts (Table 4) were significantly reduced in all cases (*p* < 0.001) but not in BorIgG+ and An+ (*p* > 0.05).

Logistic regression models (Table 5) revealed that, with the exception of An+, all the other seropositivities evaluated significantly increased (*p* < 0.05) the risk for anemia; the highest risk was detected in An+BorIgG&M+, followed by BorIgG&M+, and the lowest in An+ animals. It was also observed that BorIgG+, BorIgM+, and An+BorIgG+ seropositivity significantly increased the risk for thrombocytopenia (*p* < 0.001), and the highest risk was observed in An+BorIgG+ seropositive animals. BorIgG&M+ increased the risk for leukopenia, neutropenia, and lymphopenia (*p* < 0.001), and An+ increased the risk for leukopenia and neutropenia (*p* < 0.001). An+BorIgG&M+ seropositivity was associated with an increased risk for lymphopenia (*p* < 0.001). No other seropositivity was associated with risk for leukopenia, neutropenia, or lymphopenia (*p* < 0.001).

## 4. Discussion

In this study, we defined the impact on the hematological profile of sheep when exposed to *A. phagocytophilum* and *B. burgdorferi* s.l., separately, as well as when concurrent natural exposure to both pathogens is justified by serology.

The most prominent blood abnormalities in sheep exposed to *B. burgdorferi* s.l. were lower PCV values and PLT counts. The WBC counts were low—mainly due to low neutrophils and eosinophils counts—but increased values were also recorded for sheep in a later stage of infection (only IgG antibodies were detected). Moreover, the seropositive sheep presented an increased risk for anemia and thrombocytopenia. Our findings are in agreement with the previous observations in sheep seropositive for *B. burgdorferi* s.l. in the 2021 study by Athanasiou et al. [38].

The stage of infection was justified by the detection of IgM (BorIgM+, earlier stage), IgG (BorIgG+, later stage), and both IgM and IgG antibodies (BorIgG&M+, re-infection or intermediate stage) against *B. burgdorferi* s.l., and it seemed to slightly differentiate the hematological profile of the animal groups: PLT and WBC counts were not affected during the earlier and later stages of infection, respectively, and increased neutrophil counts were recorded only at the later stage. Moreover, lymphocyte and monocyte counts appeared to decrease only in the intermediate and earlier stages, respectively, while eosinophil counts decreased in both stages. These differences among the stages of infection are in accordance with previous observations in sheep naturally exposed to *B. burgdorferi* s.l. [38].

Single exposure to *A. phagocytophilum* in sheep was characterized by lower PCV values, lower WBC counts—mainly due to low neutrophil counts—and increased risk for leukopenia and neutropenia. The same blood abnormalities have been reported previously in sheep seropositive for *A. phagocytophilum* [21].

The reduction in the mean values of erythrocytes, hemoglobin concentration, and PCV has been reported previously; the intravascular hemolysis of erythocytes, the increased erythrocyte phagocytosis by the reticuloendothelial system, and the restricted erythropoietic activity in the bone marrow have been proposed as possible causes of these disorders [46]. Although the PCV values were low in *A. phagocytophilum* seropositive sheep, we did not find an increased risk for anemia in this group.

In the study of Abdullah et al., the researchers found increased total WBC count in *Anaplasma* seropositive sheep, and they suggested that this finding was indicative of the animals harboring the pathogen [46]—this finding is not in agreement with the results of our study. The increased risk for neutropenia described herein and elsewhere [21] is considered a key finding in *A. phagocytophilum*-infected sheep. However, discrepant results have been reported recently in a German sheep population, with none of the examined sheep presenting neutropenia [47]. Although previous studies in naturally infected sheep [21], experimentally infected cattle [48], and other animal species, such as horses and dogs [49,50], showed a low mean PLT count and thrombocytopenia, such findings were not confirmed in this study. The discrepancies found in the existing studies may reflect the different study designs and, most importantly, the fact that the sampled sheep population may be in different stages of infection. Although we cannot rule out the possibility that the locally circulating strains present different pathogenicities, data on the genetic diversity and variation in pathogenicity of the *A. phagocytophilum* strains in Greece are lacking.

Concurrent exposure to *A. phagocytophilum* and *B. burgdorferi* s.l.—justified by the detection of IgG antibodies against both agents and/or IgM antibodies against *B. burgdorferi* s.l—resulted in the most severe blood abnormalities; all the blood parameters investigated were decreased (PCV values, counts of WBCs, and all the different types of WBC). Moreover, the co-exposed animals presented an increased risk for anemia. The only differences detected between the two groups of animals were the increased risks for thrombocytopenia and lymphopenia in the An+BorIgG+ and An+BorIgG&M+ groups, respectively.

The pathogenesis of co-infection by *A. phagocytophilum* and *B. burgdorferi* s.l. in sheep most likely involves multiple mechanisms that, to the best of our knowledge, have not been investigated before. The antigenic variability associated with strain-polymorphism [30], the aspects of transmission, host and cell invasion, immune responses, and pathogen multiplication and dissemination may play a role [4]. The results of our study show that natural co-exposure of sheep to *A. phagocytophilum* and *B. burgdorferi* s.l. leads to more severe blood abnormalities compared with exposure to each pathogen alone. It seems that co-exposure enhances the pathogenic processes, but we cannot conclude on the background mechanisms. Previous studies suggested that when several pathogens infect a host, the responses may be diverse; this allows for synergistic actions and provides a greater opportunity for either pathogen to escape initial immune surveillance [4,51].

In humans, co-infection by *A. phagocytophilum* and *B. burgdorferi* s.l. leads to more severe morbidity and a longer duration of clinical signs [52]. Moreover, it results in a confusing mixture of manifestations, making diagnosis challenging [53]. Similarly, in a 2001 study, Thomas et al. showed that co-infection in mice modulated host immune responses and resulted in higher bacterial loads, more severe disease, and even increased pathogen transmission to the tick vector. The authors suggested that the reduced interleukin-12 (IL-12), gamma interferon (IFN-gamma), and tumor necrosis factor alpha levels, the increased IL-6 levels, and the reduced IFN-gamma receptor expression on macrophages—which implies a decrease in phagocyte activation—were the mechanisms behind immune response modulation [54]. In another study, the researchers found that previous infection with *A. phagocytophilum* had an effect on subsequent infection with *B. burgdorferi* s.l. Co-infection did not significantly impair antibody responses to either pathogen, but co-infected mice harbored larger populations of *B. burgdorferi* s.l. in several tissues, and by some means of immunomodulation, *B. burgdorferi* s.l. persisted longer in particular tissues [55].

The previous studies also emphasized the possible role of the immunosuppressive nature of *A. phagocytophilum* in the outcome and duration of *B. burgdorferi* s.l. infection. *Anaplasma phagocytophilum* functionally impairs neutrophils, which are important cells in the early defense against *B. burgdorferi* s.l. infection; studies have shown that *B. burgdorferi* s.l. spirochetes are susceptible to phagocytosis [56]. It has been suggested that the impairment of this innate response, together with the overall immunosuppression, may explain the increase in *B. burgdorferi* s.l. population distribution in co-infected mice [55].

An in vitro study suggested a mechanism that may contribute to increased blood and tissue spirochete loads. The researchers showed that in the presence of *A. phagocytophilum*-infected neutrophils, more *B. burgdorferi* spirochetes were able to cross endothelial cells without affecting endothelial cell integrity; the *A. phagocytophilum*-infected neutrophils released endothelial cell-derived matrix metalloproteases, cytokines, and chemokines, which increased vascular permeability and facilitated trans-endothelial cell migration of *B. burgdorferi*, thus allowing for invasion into tissues [57].

Based on the results of this study, we cannot draw any conclusions on the possible pathogenesis of co-infection by *A. phagocytophilum* and *B. burgdorferi* s.l. in sheep. However, the hematological profile of sheep after a single natural exposure to each pathogen showed some characteristics: low PCV values and PLT counts in the case of *B. burgdorferi* s.l. seropositive sheep and low PCV values, low WBC counts, and increased risk for leukopenia and neutropenia in the case of *A. phagocytophilum* seropositive sheep. Natural co-exposure resulted in the most severe blood abnormalities; all the blood parameters decreased, and the sheep presented an increased risk for anemia. This finding suggests that natural co-exposure to *A. phagocytophilum* and *B. burgdorferi* s.l. in sheep enhances the pathogenic processes, and more studies are needed to clarify the possible background mechanisms.

## Figures and Tables

**Table 1:** The allocation of the sampled animals into seven groups based on their single or combined presence of antibodies (seropositivity) against *A. phagocytophilum* and *B. burgdorferi*.

Groups	*B. burgdorferi*		*A. phagocytophilum*
	IgM	IgG	IgG
BorIgM+	+	−	−
BorIgG+	−	+	−
BorIgG&M+	+	+	−
An+BorIgG+	−	+	+
An+BorIgG&M+	+	+	+
An+	−	−	+
Control	−	−	−

**Table 2:** Linear regression coefficients (Standard Error, SE) of each single or combined presence of antibodies (seropositivity) on packed cell volume (PCV) and platelet (PLT) count.

Seropositivity	PCV	PLTs
BorIgM+	−5.03 (0.81) ***	−19,157 (34,669) ^NS^
BorIgG+	−4.97 (0.66) ***	−142,795 (28,295) ***
BorIgG&M+	−8.53 (0.95) ***	−356,990 (40,750) ***
An+BorIgG+	−4.81 (0.81) ***	−184,045 (34,669) ***
An+BorIgG&M+	−9.23 (0.83) ***	−408,285 (35,711) ***
An+	−2.10 (1.00) *	−22,733 (42,808) ^NS^

*** *p* < 0.001; * *p* < 0.05; ^NS^: non-significant; BorIgM+: *Borrelia* IgM seropositive; BorIgG+: *Borrelia* IgG seropositive; BorIgG&M+: *Borrelia* IgM and IgG seropositive; An+BorIgG+: *Anaplasma* IgG seropositive and *Borrelia* IgG seropositive; An+BorIgG&M+: *Anaplasma* IgG seropositive and *Borrelia* IgG and IgM seropositive; An+: *Anaplasma* IgG seropositive.

**Table 3:** Linear regression coefficients (SE) of each single or combined presence of antibodies (seropositivity) on white blood cells (WBC), neutrophils, and lymphocytes count.

Seropositivity	WBC	Neutrophils	Lymphocytes
BorIgM+	−1184 (517.835) *	−773 (294) **	−232 (334) ^NS^
BorIgG+	685 (422) ^NS^	712 (240) **	10 (273) ^NS^
BorIgG&M+	−3804 (608) ***	−1309 (346) ***	−2412 (393) ***
An+BorIgG+	−2040 (517) ***	−724 (294) *	−923 (334) **
An+BorIgG&M+	−6466 (533) ***	−3165 (303) ***	−2937 (344) ***
An+	−2513 (639) ***	−2058 (363) ***	−536 (413) ^NS^

*** *p* < 0.001; ** *p* < 0.01; * *p* < 0.05; ^NS^: non-significant; BorIgM+: *Borrelia* IgM seropositive; BorIgG+: *Borrelia* IgG seropositive; BorIgG&M+: *Borrelia* IgM and IgG seropositive; An+BorIgG+: *Anaplasma* IgG seropositive and *Borrelia* IgG seropositive; An+BorIgG&M+: *Anaplasma* IgG seropositive and *Borrelia* IgG and IgM seropositive; An+: *Anaplasma* IgG seropositive.

**Table 4:** Linear regression coefficients (SE) of each single or combined presence of antibodies (seropositivity) on monocytes and eosinophil count.

Seropositivity	Monocytes	Eosinophils
BorIgM+	−77 (29) *	−100 (27) ***
BorIgG+	−10 (23) ^NS^	−27 (22) ^NS^
BorIgG&M+	10 (34) ^NS^	−127 (32) ***
An+BorIgG+	−108 (29) ***	−282 (27)***
An+BorIgG&M+	−122 (30) ***	−241 (28) ***
An+	46 (36) ^NS^	36 (34) ^NS^

*** *p* < 0.001; * *p* < 0.05; ^NS^: non-significant; BorIgM+: *Borrelia* IgM seropositive; BorIgG+: *Borrelia* IgG seropositive; BorIgG&M+: *Borrelia* IgM and IgG seropositive; An+BorIgG+: *Anaplasma* IgG seropositive and *Borrelia* IgG seropositive; An+BorIgG&M+: *Anaplasma* IgG seropositive and *Borrelia* IgG and IgM seropositive; An+: *Anaplasma* IgG seropositive.

**Table 5:** Odds ratios for the presence of anemia, thrombocytopenia, leukopenia, neutropenia, and lymphocytopenia according to the serological status of the sampled animals.

Seropositivity	Anemia	Thrombocytopenia	Leukopenia	Neutropenia	Lymphocytopenia
BorIgM+	12.21 ***	10.38 ***	1.08 ^NS^	0.00 ^NS^	0.00 ^NS^
BorIgG+	16.14 ***	20.75 ***	0.60 ^NS^	3.86 ^NS^	0.00 ^NS^
BorIgG&M+	103.75 ***	0.00 ^NS^	35.00 ***	42.50 ***	28.00 ***
An+BorIgG+	30.18 ***	41.50 ***	3.50 ^NS^	0.00 ^NS^	0.00 ^NS^
An+BorIgG&M+	498.00 ***	0.00 ^NS^	0.00 ^NS^	0.00 ^NS^	30.33 ***
An+	6.92 *	1.38 ^NS^	21.78 ***	19.32 ***	4.00 ^NS^

*** *p* < 0.001; * *p* < 0.05; ^NS^: non-significant; BorIgM+: *Borrelia* IgM seropositive; BorIgG+: *Borrelia* IgG seropositive; BorIgG&M+: *Borrelia* IgM and IgG seropositive; An+BorIgG+: *Anaplasma* IgG seropositive and *Borrelia* IgG seropositive; An+BorIgG&M+: *Anaplasma* IgG seropositive and *Borrelia* IgG and IgM seropositive; An+: *Anaplasma* IgG seropositive.

## Data Availability

The data presented in this study are available on request from the corresponding author. The data are not publicly available due to further processing for other studies.
